# In-House Packed Porous Graphitic Carbon Columns for Liquid Chromatography-Mass Spectrometry Analysis of *N-*Glycans

**DOI:** 10.3389/fchem.2021.653959

**Published:** 2021-06-11

**Authors:** Clifford Young, Mark R. Condina, Matthew T. Briggs, Edward S. X. Moh, Gurjeet Kaur, Martin K. Oehler, Peter Hoffmann

**Affiliations:** ^1^ Future Industries Institute, University of South Australia, Adelaide, SA, Australia; ^2^ Department of Molecular Sciences, Macquarie University, Sydney, NSW, Australia; ^3^ Institute for Research in Molecular Medicine (INFORMM), Universiti Sains Malaysia, Pulau Pinang, Malaysia; ^4^ Department of Gynaecological Oncology, Royal Adelaide Hospital, Adelaide, SA, Australia

**Keywords:** *N*-glycan, porous graphitic carbon, liquid chromatography, mass spectrometry, hypercarb

## Abstract

Protein glycosylation is a common post-translational modification that modulates biological processes such as the immune response and protein trafficking. Altered glycosylation profiles are associated with cancer and inflammatory diseases, as well as impacting the efficacy of therapeutic monoclonal antibodies. Consisting of oligosaccharides attached to asparagine residues, enzymatically released *N-*linked glycans are analytically challenging due to the diversity of isomeric structures that exist. A commonly used technique for quantitative *N-*glycan analysis is liquid chromatography-mass spectrometry (LC-MS), which performs glycan separation and characterization. Although many reversed and normal stationary phases have been utilized for the separation of *N-*glycans, porous graphitic carbon (PGC) chromatography has become desirable because of its higher resolving capability, but is difficult to implement in a robust and reproducible manner. Herein, we demonstrate the analytical properties of a 15 cm fused silica capillary (75 µm i.d., 360 µm o.d.) packed in-house with Hypercarb PGC (3 µm) coupled to an Agilent 6550 Q-TOF mass spectrometer for *N-*glycan analysis in positive ion mode. In repeatability and intermediate precision measurements conducted on released *N-*glycans from a glycoprotein standard mixture, the majority of *N-*glycans reported low coefficients of variation with respect to retention times (≤4.2%) and peak areas (≤14.4%). *N-*glycans released from complex samples were also examined by PGC LC-MS. A total of 120 *N-*glycan structural and compositional isomers were obtained from formalin-fixed paraffin-embedded ovarian cancer tissue sections. Finally, a comparison between early- and late-stage formalin-fixed paraffin-embedded ovarian cancer tissues revealed qualitative changes in the α2,3- and α2,6-sialic acid linkage of a fucosylated bi-antennary complex *N-*glycan. Although the α2,3-linkage was predominant in late-stage ovarian cancer, the alternate α2,6-linkage was more prevalent in early-stage ovarian cancer. This study establishes the utility of in-house packed PGC columns for the robust and reproducible LC-MS analysis of *N-*glycans.

## Introduction

In humans, the glycosylation machinery responsible for the orchestrated glycosylation of proteins is encoded by approximately 700 human genes (Schjoldager et al., 2020). Glycosylation has the ability to modify the physicochemical properties and biological functions of proteins (Lis and Sharon, 1993), which influences many cellular processes such as the immune response (Rudd et al., 2001) and protein trafficking (Varki, 2017). Alterations in glycosylation patterns have been reported in inflammatory and congenital diseases, as well as cancer (Reily et al., 2019). Consequently, the development of analytical tools that discriminate glycosylation profiles is important from a clinical perspective and for characterizing the role of glycosylation in the efficacy and safety of protein therapeutics (Liu, 2015).

There are two main types of protein glycosylation: *N-*linked glycosylation, where oligosaccharides are attached to an asparagine residue; and *O-*linked glycosylation, where sugars are attached to a serine or threonine residue. A general workflow for glycan analysis involves PNGase F incubation to enzymatically release the *N-*glycans, where they are subsequently reduced and cleaned up prior to liquid chromatography-mass spectrometry (LC-MS) (Jensen et al., 2012). Chemical derivatization of glycans is also often employed following PNGase F digestion to aid their detection in LC-MS (Ruhaak et al., 2010). Overall, many methodologies exist to obtain both qualitative and quantitative glycomics data, which reflects the immense diversity and hydrophilic nature of *N-*glycans.

Although no single chromatographical technique is currently capable of comprehensive glycan separation, hydrophilic interaction chromatography (HILIC) and porous graphitic carbon (PGC) chromatography are often performed prior to mass spectrometry (MS) analysis. In the presence of a high organic mobile phase, HILIC permits glycan adsorption on a hydrophilic stationary phase by hydrogen bonding, ionic interactions, and dipole-dipole interactions (Wuhrer et al., 2009). Although HILIC allows many *N-*glycans to be sufficiently resolved, PGC chromatography has become useful for its ability to separate underivatized *N-*glycan isomeric structures (Jensen et al., 2012). This is despite PGC possessing unique adsorption and retention mechanisms, which are thought to be based upon dispersive interactions and a polar retention effect on graphite (Hanai, 2003; West et al., 2010). Resolved *N-*glycan isomers are then subjected to mass spectrometric analysis, where the mass of the glycan is obtained and glycan dissociation performed during tandem mass spectrometry (MS/MS) to generate fragment ions that reveal isomer specific structural information (Han and Costello, 2013).

As capillary PGC columns have become difficult to obtain commercially, the possibility of constructing fused silica PGC columns with narrower internal diameters was investigated. A major benefit from column miniaturization would be the increase in analyte sensitivity resulting from electrospray ionization performed at lower flow rates (Emmett and Caprioli, 1994). In this paper, we produced in-house packed PGC columns to perform LC-MS analysis of *N-*glycans from various sample types with high sensitivity and reproducibility in positive ion mode. The technological setup was evaluated on a *N-*glycan mixture prepared from known glycoproteins (human neutrophil elastase, human serum IgG, bovine RNase B, and bovine fetuin) before performing the analysis on released *N-*glycans from complex biological samples, such as early- and late-stage formalin-fixed paraffin-embedded (FFPE) ovarian cancer tissues. We detected a total of 120 *N*-glycans (including structural and compositional isomers) from these cancer tissues, which was considerably more than the 42 isomers obtained in negative ion mode by our group using a shorter commercially available PGC column (Briggs et al., 2019). A comparison of the two cancer stages also found major changes in the distribution of an α2,3- and α2,6-sialic acid linkage to a fucosylated bi-antennary complex *N-*glycan. These results together demonstrate the analytical power of self-manufactured PGC columns for the LC-MS analysis of *N-*glycans, which is consistent with reports from other groups (Thomsson et al., 2010; She et al., 2020).

## Materials and Methods

### Glycoprotein Standard Mixture Preparation

Human neutrophil elastase (300 µg), human serum IgG (300 µg), bovine RNase B (300 µg), and bovine fetuin (600 µg) were heat denatured for 10 min at 95°C. After pooling the glycoproteins, 25 µL of PNGase F (Promega) was added for overnight digestion with intermittent mixing. The sample was aliquoted into six eppendorfs for protein precipitation using a mixture of chloroform/methanol/water in a 1:4:4 ratio. The methanol layer was dried and deaminated with 100 mM ammonium acetate (pH 5). After drying, the standards were reduced with 40 µL of 1 M sodium borohydride in 50 mM potassium hydroxide. The samples were neutralized, recombined, and diluted five-fold with water prior to clean up using a pre-equilibrated StrataX-C18 column. The flowthrough was directly loaded onto a pre-equilibrated ENVI-Carb SPE column and washed with water. The analytes were eluted with 40% acetonitrile in 0.05% trifluoroacetic acid and aliquoted into fresh eppendorfs before drying. Each eppendorf contained an amount of released *N-*glycans from 100 µg equivalent of the glycoprotein mixture. When required, each eppendorf was reconstituted in 50 µL water, with 2 µL of the glycoprotein standard mixture loaded for LC-MS analysis.

### Formalin-Fixed Paraffin-Embedded Ovarian Cancer Tissue Preparation

The use of FFPE ovarian cancer tissue was reviewed and approved by the Human Ethics Committee at the University of South Australia. The patients provided their written informed consent to participate in this study. After obtaining ethics approval, early- (*n* = 3) and late-stage (*n* = 3) FFPE ovarian cancer tissue blocks were collected (Supplementary Table S1). FFPE blocks were sectioned and mounted as previously described (Briggs et al., 2017). Consecutive sections were haematoxylin and eosin stained and tumor-specific regions were annotated. After dewaxing, approximately 1 cm^2^ FFPE ovarian cancer tissue sections on PEN membrane slides were manually dissected, antigen retrieved, rehydrated, digested with PNGase F, reduced, and cleaned up as previously described (Briggs et al., 2017). After the eluates were dried, samples were reconstituted in 10 µL water, with 2 µL injected for LC-MS analysis.

### Frit Fabrication and Porous Graphitic Carbon Column Packing

Fritted 25 cm fused silica capillaries (75 µm i.d., 360 µm o.d. from Polymicro Technologies, Phoenix, AZ) were prepared by inserting one end into a mixture of 75% Kasil 1624 and 25% formamide for 3 s before leaving the capillaries in a heating block at 95°C for overnight polymerization (frit side down). The frit was cut to 1 mm prior to column packing. A 10 mg/mL slurry of 3 µm Hypercarb PGC (Thermo Scientific) in methanol was used to pack a fritted capillary in a Pressure Injection Cell (Next Advance, Troy, NY). The first 5 cm of the PGC column was packed at a nitrogen pressure of 25 bar, which was increased to 100 bar to obtain a 20 cm packed column. The PGC column was cut to the desired length (15 cm) immediately prior to LC-MS experiments.

### Porous Graphitic Carbon Liquid Chromatography-Mass Spectrometry

*N-*glycan mixtures were analyzed on a 1290 Infinity II LC System (Agilent Technologies, Palo Alto, CA) connected to a 6550 iFunnel Q-TOF mass spectrometer (Agilent Technologies). An uncoated emitter tip was attached to the end of the PGC column with a conductive ferrule and stainless steel union in line with the MS inlet, with additional cables attached in several locations to ensure electrical grounding of the PGC column. Mobile phases for chromatography consisted of buffer A (5 mM ammonium formate) and buffer B (95% acetonitrile in 5 mM ammonium formate). *N-*glycan samples were directly loaded onto the 15 cm in-house packed Hypercarb column (3 µm, 75 µm i.d., 360 µm o.d.) in buffer A for 9 min at 0.06 mL/min, which is approximately 800 nL/min after the flow was split by an Infinity UHPLC Nanodapter (Agilent Technologies). Elution of *N-*glycans was performed at 0.06 mL/min using a 70 min gradient composed of: 2–12% B (30 min), 12–60% B (22 min), 60–95% B (1 min), 95% B (2 min), 95–2% B (1 min), and 2% B (14 min). MS (*m/z* 300–3000) and MS/MS scans (*m/z* 100–3000) were obtained in positive ion mode at an acquisition rate of 333 ms/scan. Source parameters were specified as: gas temperature, 130°C; drying gas, 15 L/min; capillary voltage, 1800 V; precursor isolation width, 4 amu; fragmentor voltage 125 V; and octopole RF V (pp) 750 V. MS/MS parameters were set to three max MS/MS per cycle, an absolute threshold of 0.01% (500 counts absolute threshold), an active exclusion of one spectrum released after 0.5 min, and precursors sorted by abundance only. Collision energy settings for MS/MS scans were performed as described (Table 1). The PGC column was flushed with 95% methanol between experiments to maintain column performance (Bapiro et al., 2016).

**TABLE 1:** MS/MS collision energy settings for different mass-to-charge ratios (*m/z*) and charge states (*z*).

	Collision energy (V)			
*m/z*	*z* = 1	*z* = 2	*z* = 3	*z* > 3
300	34	17	13	17
500	40	21	16	21
700	43	22	22	24
1000	45	25	29	26
2000	50	30	35	30

### Data Analysis

The identification and characterization of *N-*glycan structures was based upon criteria established in a previous study (Briggs et al., 2019), which involved the manual assignment and annotation of MS/MS data using GlycoWorkbench software (Ceroni et al., 2008).

## Results

**FIGURE 1:**
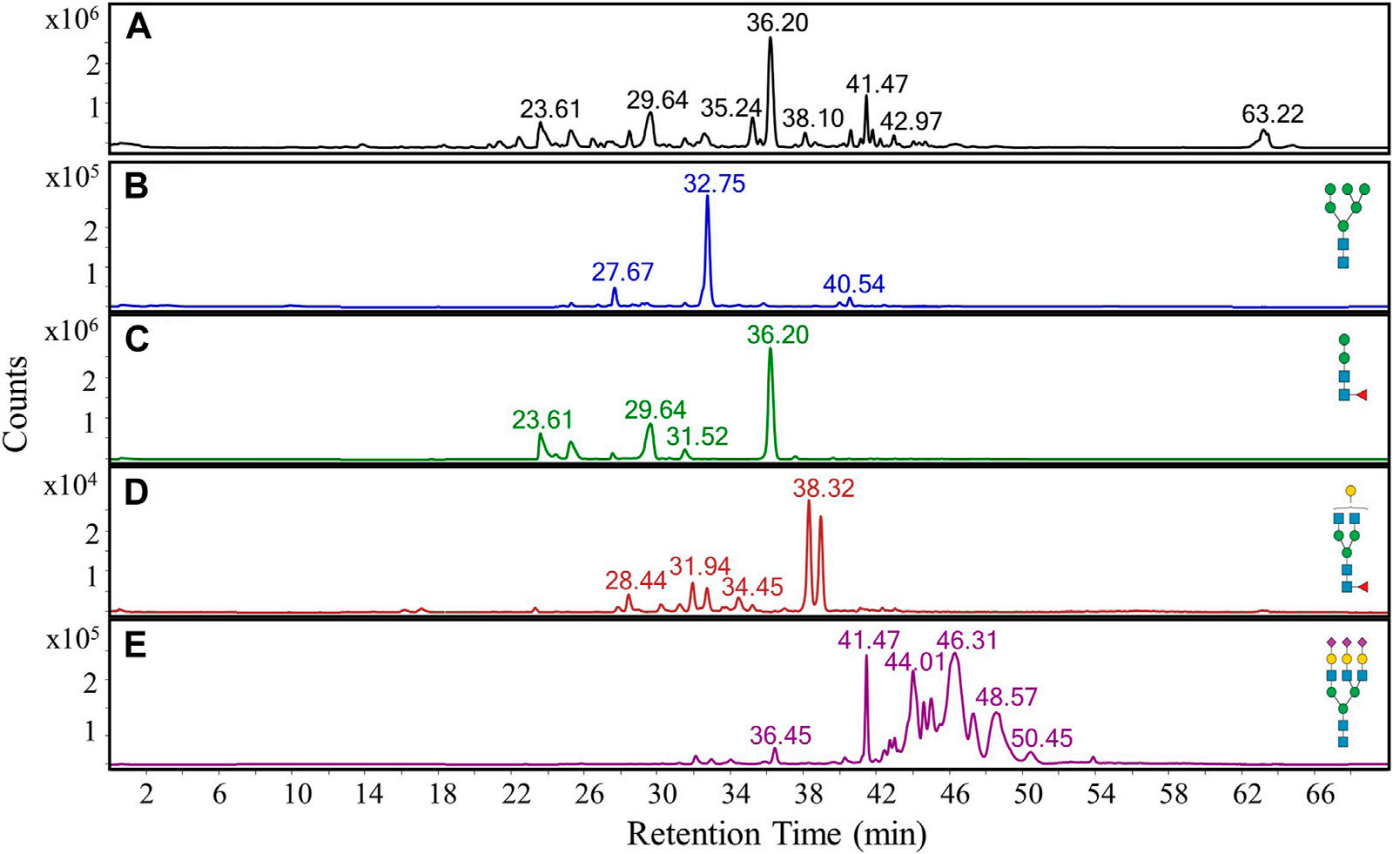
PGC LC-MS separation and detection of *N-*glycans released from an in-house glycoprotein standard mixture. **(A)** Representative base peak chromatogram of *N-*glycans. **(B**–**E)** Extracted ion chromatograms of four representative *N-*glycans, namely an oligomannose structure **(B)**, a fucosylated paucimannose structure **(C)**, a fucosylated bi-antennary complex structure **(D)**, and a tri-antennary sialylated structure **(E)**.

When used in conjunction with electrospray ionization, PGC columns are particularly prone to reproducibility issues. Spray instability and analyte retention time shifts often occur because of redox reactions that originate from electrical current travelling through the conductive stationary phase (Törnkvist et al., 2004). These problems can be mitigated by rerouting any residual current away from the PGC column through grounding points. To this end, we mounted grounding cables and investigated whether a stable spray could be established with different buffers. We evaluated 10 mM ammonium bicarbonate (Jensen et al., 2012) and then 0.04% ammonium hydroxide (Thomsson et al., 2010) as they were successfully used in negative ion mode PGC LC-MS. However, spray formation was not possible with 10 mM ammonium bicarbonate and despite initial success with 0.04% ammonium hydroxide, the spray performance deteriorated over consecutive blank PGC LC-MS runs (data not shown). However, the use of 5 mM ammonium formate in positive ion mode resulted in a stable spray and emitter fouling was also not observed. Therefore, these mobile phase conditions were incorporated into all subsequent LC-MS experiments. The chromatographical capabilities of the 15 cm fused silica column packed with 3 µm Hypercarb was evaluated using *N-*glycans released from a glycoprotein standard mixture using a 70 min gradient. Most analytes eluted with retention times ranging from 20 to 55 min (Figure 1A).

Furthermore, it was possible to elucidate specific *N-*glycan families from their different retention time ranges. While oligomannose (Figure 1B) and paucimannose (Figure 1C) structures eluted between 22 and 42 min, bi-antennary complex *N-*glycan structures (Figure 1D) were predominantly observed at a later retention time window extending from 27 to 40 min. Tri-antennary sialylated structures (Figure 1E) eluted at a later time period between 30 and 54 min, with the detection of many isomeric sialoglycans. This complexity is largely due to the resolution of isomeric species resulting from α2,3- or α2,6-linkages between each sialic acid and galactose.

### Repeatability and Intermediate Precision Analysis of In-House Packed Porous Graphitic Carbon Columns

**FIGURE 2:**
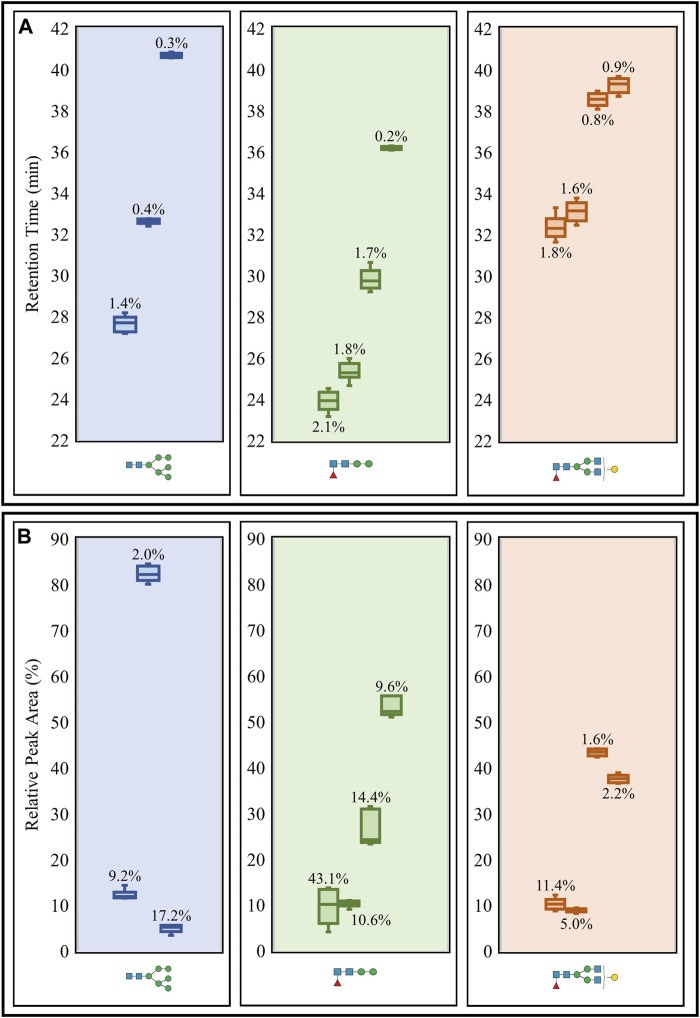
Repeatability study of retention times **(A)** and relative peak areas **(B)** of three representative *N-*glycans (including isomeric structures) released from the glycoprotein standard mixture. Box plots and CVs (annotated) were calculated for each *N-*glycan representative detected from consecutive sextuplicate PGC LC-MS runs. Raw retention time data (Supplementary Table S2) and raw relative peak area data (Supplementary Table S3) are available.

Repeatability of the PGC LC-MS setup was assessed from six consecutive replicate runs of *N-*glycans released from the same glycoprotein standard mixture. Coefficients of variation (CVs) were calculated for the retention times and relative peak areas of three representative *N-*glycans and their respective isomeric structures (Figure 2). Retention time CVs were generally low (Figure 2A), with the highest measurement reaching only 2.1%. However, the run-to-run variability of the relative peak areas was more inconsistent (Figure 2B). Despite an oligomannose structure and a paucimannose isomer yielding CVs of 17.2 and 43.1%, respectively, the remaining CVs were below 15%.

**FIGURE 3:**
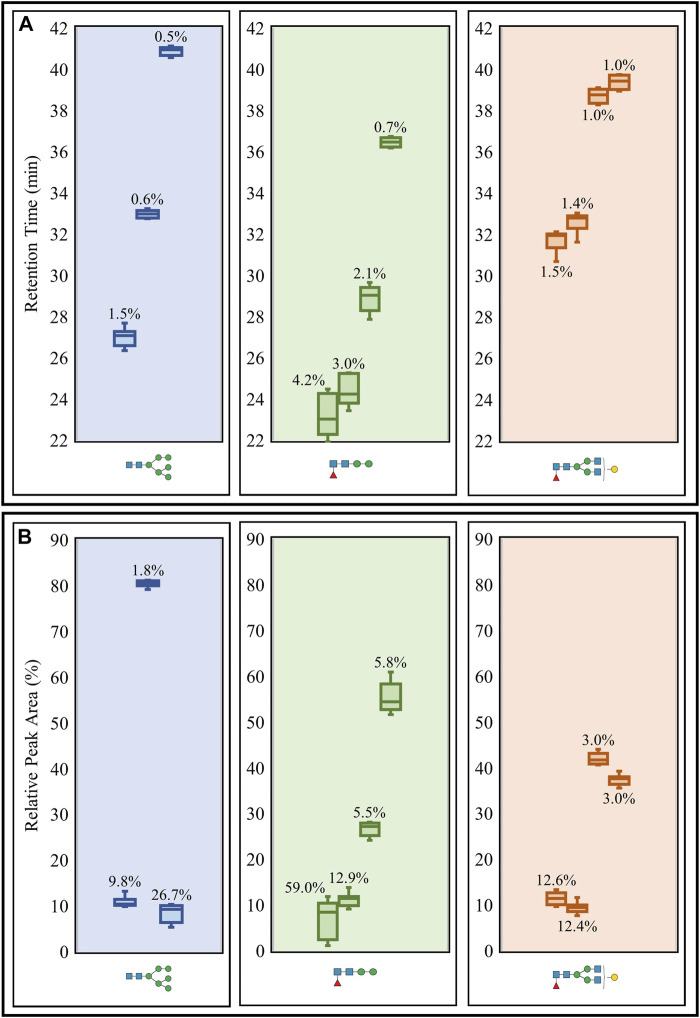
Intermediate precision study of retention times **(A)** and relative peak areas **(B)** of three representative *N-*glycans (including isomeric structures) released from the glycoprotein standard mixture. Box plots and CVs (annotated) were calculated for each *N-*glycan representative detected from triplicate PGC LC-MS runs performed over three days. Raw retention time data (Supplementary Table S4) and raw relative peak area data (Supplementary Table S5) are available.

Intermediate precision was then evaluated using triplicate LC-MS runs of the aforementioned *N-*glycans obtained on three separate days. CVs for the retention times and relative peak areas of the same three representative *N-*glycans and their respective isomeric structures were used for the assessment (Figure 3). The retention time CVs were all low, reaching a maximum of 4.2% (Figure 3A), while many of the relative peak areas were below 13% (Figure 3B). The same oligomannose and paucimannose structures that possessed high peak area CVs in the repeatability study also registered high CVs (26.7 and 59%, respectively) in the intermediate precision study. It should be noted that the high CVs were generally observed for low abundance *N-*glycans.

### Porous Graphitic Carbon Liquid Chromatography-Mass Spectrometry Analysis of Early- and Late-Stage Formalin-Fixed Paraffin-Embedded Ovarian Cancer Tissues

After achieving robust and reproducible chromatographical separations of many released *N*-glycans from the glycoprotein standard mixture, the capability of the PGC LC-MS apparatus to analyze *N*-glycans released from early- and late-stage FFPE ovarian cancer tissues was assessed. Although 26 distinct *N-*glycan structures were identified and characterized from microdissected classified tumor-specific regions (Supplementary Table S6), a total of 120 *N-*glycan structural and compositional isomers could be determined.

**FIGURE 4:**
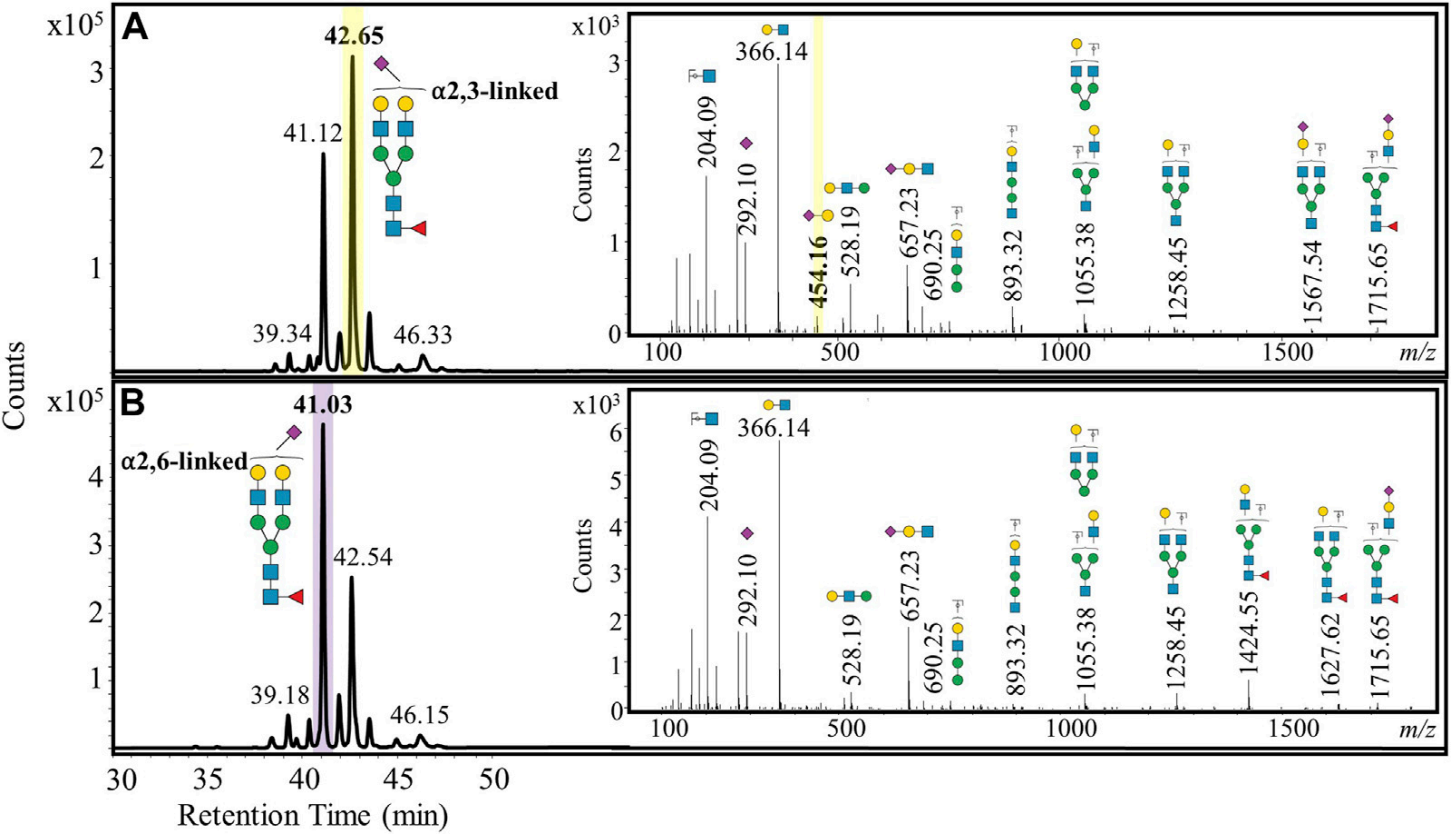
Representative extracted ion chromatograms of a doubly positively charged monoisotopic species (*m/z* 1040.89), which was identified and characterized as a fucosylated bi-antennary complex *N-*glycan that was singly sialylated. The sialylation was predominantly α2,3-linked for late-stage ovarian cancer **(A)** and α2,6-linked for early-stage ovarian cancer **(B)**.

We then investigated whether these *N-*glycans from early- and late-stage FFPE ovarian cancer samples exhibited stage-specific differences. A fucosylated bi-antennary complex *N-*glycan that was singly sialylated showed a marked distribution change in the sialic acid linkage, where the α2,3-linked isomer was consistently more prominent than the α2,6-linked isomer in late-stage ovarian cancer patient samples (Figure 4A). A diagnostic b_2_ fragment ion (*m/z* 454.16) corresponding to the α2,3-linked isomer (She et al., 2020) was detected in MS/MS spectra from late-stage ovarian cancer patients, confirming the presence of this specific isomer. However, in early-stage ovarian cancer samples, the α2,6-sialic acid linked isomer was more dominant than the α2,3-sialic acid linked isomer (Figure 4B).

## Discussion

PGC is a useful stationary phase for glycan separation as it permits stronger retention of native and small glycan species, presumably through dispersive interactions and a polar retention effect on graphite (Hanai, 2003; West et al., 2010). The ability of PGC to resolve isomers of *N-*glycans is generally superior to other separation strategies, including HILIC (Jensen et al., 2012; Ashwood et al., 2019). The coupling of PGC chromatography with MS has been successfully adopted for the characterization of released *N-*glycans from complex samples, such as ovarian cancer cell lines (Anugraham et al., 2014) and tissues (Briggs et al., 2019; Anugraham et al., 2017). Nonetheless, the widespread adoption of PGC LC-MS for isomeric glycan separations has been hampered due to concerns over robustness and reproducibility (Veillon et al., 2017). Improvised strategies that have overcome the deficiencies associated with PGC chromatography of various analytes include: electrical grounding of the column (Pabst and Altmann, 2008), flushing the column between sample runs with 95% methanol (Bapiro et al., 2016), or performing column washes with two 50% acetonitrile solutions at an acidic and basic pH (Pabst and Altmann, 2008). We incorporated several of these aspects to promote good spray formation and mitigate conditions that could cause alterations in the chromatographic properties of PGC.

A *N-*glycan standard prepared from several glycoproteins was used to evaluate the stability of the PGC LC-MS configuration. We observed *N*-glycan ions predominantly in the protonated form among the multiple charge states, but also the formation of ammonium and sodium adducts (data not shown). The combined intensity of the ammonium and sodium adducts was estimated to be less than 10% of the observed protonated species, indicating adduct formation was negligible. Multiple injections were conducted to assess repeatability and intermediate precision for the major subclasses of *N*-glycans. Although the separation and detection of oligomannose, fucosylated paucimannose, and fucosylated bi-antennary complexes were performed to an acceptable level, many of the tri-antennary sialylated structures were not (Figure 1E). While ample separation would be harder to achieve with the presence of many isomeric structures, their suboptimal elution could be improved upon by altering the ionic strength and pH of the mobile phases, as these factors can considerably alter the elution profile and peak shape for sialyated glycans (Pabst and Altmann, 2008). It should be noted that such an approach to improve the elution of these structures might come at the expense of lower ionization efficiency and/or spray instability, which would therefore affect overall peak area quantification.

Statistical analysis of the three non-sialylated structures revealed retention time CVs lower than 5% in the repeatability (Figure 2A) and intermediate precision (Figure 3A) tests, which indicates a high level of precision with respect to instrument performance. Investigating these aspects over longer periods of time would provide a more definitive overview on the true reproducibility of the PGC LC-MS apparatus. Apart from two low abundant *N-*glycan structures (oligomannose and paucimannose) reporting high relative peak area CVs in the repeatability (Figure 2B) and intermediate precision (Figure 3B) experiments, the remaining CVs were generally below 15%. We hypothesized that the high variability associated with the paucimannose isomer could originate from the in-source fragmentation of another *N-*glycan, as we did not expect to see so many isomers. However, at the observed retention time for the paucimannose isomer, many other co-eluting glycan species were present. Although we could not confirm whether this observed isomer was the result of in-source fragmentation, further work involving the use of alternative MS instrumentation or conducting in-source fragmentation experiments on specific glycan standards would greatly assist in determining the origin of the observed high variability.

The vast majority of PGC LC-MS analyses use negative ion mode because of the improved detection of sialylated *N*-glycans and associated isomers (Nguyen-Khuong et al., 2018; Harvey, 2020). We previously utilized capillary negative ion PGC LC-MS to characterize *N*-glycan changes in ovarian cancer progression and found complex bi-antennary *N*-glycans to be unique markers for disease progression in late-stage ovarian cancer tissue (Briggs et al., 2019). When the released *N*-glycans from the same ovarian cancer tissues were analyzed on our current PGC LC-MS, all 26 previously structurally characterized glycans were observed, including the sialylated species (Supplementary Table S6). This indicates that positive ion mode permits the observation of sialylated *N*-glycans, which is consistent with an earlier report (She et al., 2020). Moreover, a total of 120 structural and compositional isomers were characterized in the current study, which is a considerable improvement over the 42 *N-*glycan isomers detected in negative ion mode using a commercial Hypercarb analytical column. Other research groups that have characterized released *N*-glycans from ovarian cancer cell lines or tissues with negative ion mode PGC LC-MS have found up to 55 *N*-glycan structures and approximately 70 structural isomers (Anugraham et al., 2014; Anugraham et al., 2017). An explanation for the numerical differences in *N*-glycan structures and associated isomers between these two studies and ours is likely to be multifactorial, with the outcome influenced by dissimilarities in: the amount of starting material, PGC column dimensions, solvent composition and flow rates for chromatographical separation, MS ionisation mode, and the extent of in-source fragmentation among different mass spectrometers.

Sialyltransferases are the glycoenzymes responsible for attaching sialic acid to nascent *N-*glycans. It is well established that sialyltransferases can lead to hypersialylated cell surface *N-*glycans in breast (Scott and Drake, 2019) and prostate (Munkley et al., 2016) cancers. ST3GAL1, the sialyltransferase gene responsible for the attachment of sialic acid in the α2,3 position, has been reported to be upregulated in ovarian cancer tissue and in the SKOV-3 and OVCAR3 cell lines (Wu et al., 2018). These findings validate our observation that the α2,3-linked sialylated isomer was expressed more than the α2,6-linked sialylated isomer in late-stage ovarian cancer patients with respect to a mono-sialylated mono-fucosylated bi-antennary complex *N-*glycan (Figure 4A). Conversely, the early-stage ovarian cancer patients revealed that the α2,6-linked sialylated isomer was more abundant than the α2,3-linked sialylated isomer (Figure 4B). To our knowledge, changes in sialic acid linkage have not been investigated in early-stage ovarian cancer tissue and would therefore warrant an investigation involving a larger patient cohort. Our finding of an increased α2,3-linked sialylated isomer in ovarian cancer tissue has also been reported in ovarian cancer disease progression from serum samples (De˘dová et al., 2019). It is also known that α2,3-linked sialylated *N-*glycans elute at later retention times on PGC columns than the corresponding α2,6-linked sialylated *N-*glycans (Palmisano et al., 2013), which was consistent with our observations.

Although there were noticeable sialic acid linkage differences between early- and late-stage ovarian cancer patients, the small sample sizes precluded any meaningful statistical analysis. Quantitative information could be obtained from larger sets of samples (ovarian cancer tissue microarrays) in combination with methodological improvements, such as the use of a *N*-glycan retention library (Abrahams et al., 2018) or a spiked dextran ladder standard of known concentration to allow the normalization of retention time and peak area shifts (Ashwood et al., 2019). Finally, since cancer antigen 125 (CA125) is an ovarian cancer biomarker that is heavily *O-*glycosylated (3700 *O-*glycosites and 249 *N-*glycosites), performing and optimizing *O-*glycan analysis in positive ion mode on the PGC LC-MS system would be of considerable biological interest (Saldova et al., 2013).

In summary, we have demonstrated the performance of a LC-MS system that utilizes in-house packed PGC columns for the analysis of released *N-*glycans in positive ion mode. For the vast majority of *N-*glycans that were detected in a glycoprotein standard mixture, low variability was observed with respect to repeatability and intermediate precision experiments. When FFPE ovarian cancer tissue sections were assessed, over 100 *N*-glycan isomeric structures (including many sialylated *N*-glycans) were detected in positive ion mode. Importantly, the analysis of FFPE tissue from early- and late-stage ovarian cancer patients revealed higher amounts of α2,6- and α2,3-linked sialic acids on a fucosylated bi-antennary complex *N-*glycan, respectively. Despite the commercial unavailability of PGC columns, we show that self-packed PGC columns are a suitable alternative.

## Data Availability

The datasets presented in this study can be found in online repositories. The names of the repository/repositories and accession number(s) can be found below: ProteomeXchange Consortium via the PRIDE (Perez-Riverol et al., 2019) partner repository, dataset identifier PXD024489.
