# Supplementary material for: In-House Packed Porous Graphitic Carbon Columns for Liquid Chromatography-Mass Spectrometry Analysis of N-Glycans
Source: Front Chem. 2021 Jun 11;9:653959. doi: 10.3389/fchem.2021.653959 (PMC8226321; doi:10.3389/fchem.2021.653959)
Supplement: Supplementary file 1 [file DataSheet1.docx]

**Supplementary Table S1:** International Federation of Gynaecology and Obstetrics stage I and stage III serous ovarian cancer patient information.

| Patient | Age | Stage | Grade | Diagnosis |
| --- | --- | --- | --- | --- |
| A | 58 | IC | 3 | Serous Ovarian Carcinoma |
| B | 46 | IC | 3 | Serous Ovarian Carcinoma |
| C | 59 | IA | 3 | Serous Ovarian Carcinoma |
| D | 66 | IIIC | 3 | Serous Ovarian Carcinoma |
| E | 47 | IIIC | 3 | Serous Ovarian Carcinoma |
| F | 60 | IIIC | 3 | Serous Ovarian Carcinoma |

**Supplementary Table S2:** Repeatability study of retention times (in minutes) for three representative *N-*glycans (including isomeric structures) released from the glycoprotein standard mixture. PGC LC-MS runs were performed in sextuplicate.

| **Glycan** | **Run 1** | **Run 2** | **Run 3** | **Run 4** | **Run 5** | **Run 6** |
| --- | --- | --- | --- | --- | --- | --- |
| 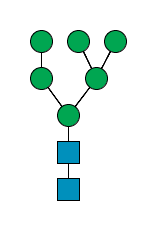 | 27.893 | 27.710 | 28.176 | 27.175 | 27.269 | 27.670 |
|  | 32.387 | 32.557 | 32.593 | 32.662 | 32.738 | 32.750 |
|  | 40.663 | 40.842 | 40.757 | 40.574 | 40.632 | 40.558 |
| 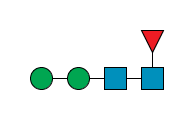 | 23.151 | 24.266 | 24.523 | 24.178 | 23.678 | 23.607 |
|  | 24.646 | 25.648 | 25.973 | 25.211 | 25.285 | 25.248 |
|  | 30.631 | 29.843 | 30.100 | 29.205 | 29.454 | 29.643 |
|  | 36.119 | 36.294 | 36.216 | 36.077 | 36.248 | 36.198 |
| 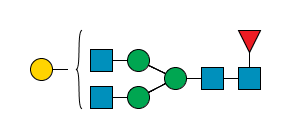 | 33.307 | 32.604 | 32.593 | 31.631 | 32.022 | 31.992 |
|  | 33.778 | 33.463 | 33.432 | 32.452 | 32.855 | 32.726 |
|  | 38.697 | 38.967 | 38.788 | 38.079 | 38.453 | 38.323 |
|  | 39.417 | 39.667 | 39.535 | 38.705 | 39.2 | 38.951 |

**Supplementary Table S3:** Repeatability study of relative peak areas for three representative *N-*glycans (including isomeric structures) released from the glycoprotein standard mixture. PGC LC-MS runs were performed in sextuplicate. Percentage totals may not equal 100% due to rounding.

| **Glycan** | **Average Retention Time** | **Run 1** | **Run 2** | **Run 3** | **Run 4** | **Run 5** | **Run 6** |
| --- | --- | --- | --- | --- | --- | --- | --- |
| 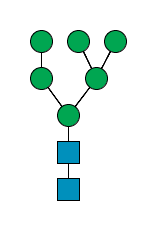 | 27.649 | 14.3% | 11.6% | 12.1% | 11.6% | 13.2% | 11.5% |
|  | 32.615 | 80.1% | 83.9% | 84.5% | 83.3% | 81.2% | 83.1% |
|  | 40.671 | 5.6% | 4.5% | 3.4% | 5.1% | 5.6% | 5.3% |
| 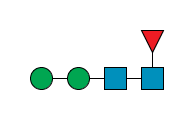 | 23.901 | 7.3% | 4.1% | 6.5% | 12.9% | 13.7% | 13.3% |
|  | 25.335 | 10.2% | 8.0% | 11.0% | 10.8% | 10.4% | 10.0% |
|  | 29.813 | 31.5% | 23.3% | 30.7% | 23.7% | 24.3% | 24.1% |
|  | 36.192 | 51.0% | 64.5% | 51.8% | 52.7% | 51.7% | 52.6% |
| 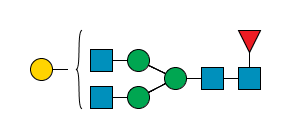 | 32.358 | 10.1% | 8.7% | 12.2% | 10.2% | 9.6% | 10.3% |
|  | 33.118 | 8.3% | 8.2% | 8.9% | 9.0% | 9.4% | 8.9% |
|  | 38.551 | 43.9% | 44.2% | 42.3% | 42.9% | 43.4% | 44.0% |
|  | 39.246 | 37.7% | 38.9% | 36.6% | 37.8% | 37.6% | 36.8% |

**Supplementary Table S4:** Intermediate precision study of retention times (in minutes) for three representative *N-*glycans (including isomeric structures) released from the glycoprotein standard mixture. Triplicate PGC LC-MS runs were performed each day over a period of three days.

|  | **Day 1** | | | **Day 2** | | | **Day 3** | | |
| --- | --- | --- | --- | --- | --- | --- | --- | --- | --- |
| **Glycan** | **Run 1** | **Run 2** | **Run 3** | **Run 1** | **Run 2** | **Run 3** | **Run 1** | **Run 2** | **Run 3** |
| 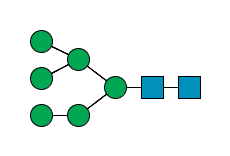 | 27.175 | 27.269 | 27.670 | 26.681 | 26.549 | 26.325 | 27.082 | 27.059 | 27.220 |
|  | 32.662 | 32.738 | 32.750 | 33.146 | 32.943 | 32.839 | 32.992 | 33.098 | 33.230 |
|  | 40.574 | 40.632 | 40.558 | 41.123 | 41.071 | 40.939 | 40.785 | 40.936 | 40.953 |
| 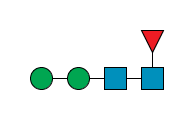 | 24.178 | 24.466 | 24.432 | 22.583 | 21.901 | 22.158 | 23.106 | 22.912 | 23.757 |
|  | 25.211 | 25.237 | 25.259 | 24.128 | 23.429 | 23.669 | 24.276 | 24.174 | 25.220 |
|  | 29.205 | 29.454 | 29.637 | 28.412 | 28.228 | 27.851 | 29.230 | 28.970 | 29.089 |
|  | 36.077 | 36.248 | 36.198 | 36.690 | 36.387 | 36.174 | 36.524 | 36.621 | 36.730 |
| 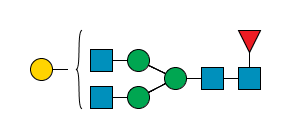 | 31.631 | 32.022 | 31.992 | 31.607 | 31.245 | 30.681 | 32.112 | 31.900 | 31.972 |
|  | 32.452 | 32.855 | 32.726 | 32.555 | 32.181 | 31.612 | 33.015 | 32.827 | 32.898 |
|  | 38.079 | 38.453 | 38.323 | 39.108 | 38.725 | 38.273 | 38.778 | 38.964 | 39.037 |
|  | 38.705 | 39.200 | 38.951 | 39.741 | 39.426 | 38.943 | 39.407 | 39.664 | 39.716 |

**Supplementary Table S5:** Intermediate precision study of relative peak areas for three representative *N-*glycans (including isomeric structures) released from the glycoprotein standard mixture. Triplicate PGC LC-MS runs were performed each day over a period of three days. Percentage totals may not equal 100% due to rounding.

|  | | **Day 1** | | | **Day 2** | | | **Day 3** | | |
| --- | --- | --- | --- | --- | --- | --- | --- | --- | --- | --- |
| **Glycan** | **Average Retention Time** | **Run 1** | **Run 2** | **Run 3** | **Run 1** | **Run 2** | **Run 3** | **Run 1** | **Run 2** | **Run 3** |
| 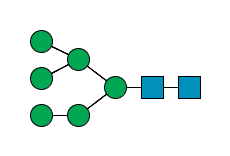 | 27.003 | 11.6% | 13.2% | 11.5% | 10.3% | 10.1% | 11.1% | 9.8% | 10.5% | 10.2% |
|  | 32.933 | 83.3% | 81.2% | 83.1% | 80.6% | 80.6% | 80.3% | 80.1% | 79.1% | 79.9% |
|  | 40.841 | 5.1% | 5.6% | 5.3% | 9.1% | 9.3% | 8.6% | 10.1% | 10.3% | 9.9% |
| 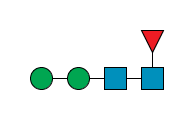 | 23.277 | 11.8% | 2.4% | 2.3% | 8.4% | 1.1% | 11.6% | 9.1% | 8.7% | 6.4% |
|  | 24.511 | 10.4% | 11.5% | 11.9% | 9.6% | 10.1% | 9.1% | 11.7% | 11.4% | 13.8% |
|  | 28.897 | 24.1% | 27.5% | 27.8% | 25.5% | 27.9% | 24.8% | 27.1% | 26.6% | 28.1% |
|  | 36.405 | 53.7% | 58.6% | 58.0% | 56.5% | 60.9% | 54.4% | 52.0% | 53.3% | 51.6% |
| 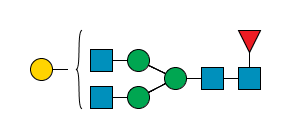 | 31.685 | 10.2% | 9.6% | 10.3% | 12.0% | 12.0% | 13.3% | 11.4% | 9.8% | 13.3% |
|  | 32.569 | 9.0% | 9.4% | 8.9% | 7.6% | 8.1% | 9.7% | 11.6% | 10.2% | 9.7% |
|  | 38.638 | 42.9% | 43.4% | 44.0% | 42.7% | 41.7% | 40.7% | 41.5% | 40.8% | 40.6% |
|  | 39.306 | 37.8% | 37.6% | 36.8% | 37.7% | 38.2% | 36.3% | 35.5% | 39.2% | 36.4% |

**Supplementary Table S6**: *N-*glycan structures identified and characterized by PGC LC-MS from FFPE tissue of early- (*n* = 3) and late-stage (*n* = 3) ovarian cancer patients. Y = Yes and N = No.

| **PGC LC-MS** | | | | | **Stage I** | | | **Stage III** | | | **Composition** | **Structure** |
| --- | --- | --- | --- | --- | --- | --- | --- | --- | --- | --- | --- | --- |
| **Number** | **Retention**  **Time (min)** | **[M+3H]^3+^** | **[M+2H]^2+^** | **[M+H]^1+^** | **A** | **B** | **C** | **D** | **E** | **F** |  |  |
| 1a | 27.98 | 467.17 | 700.26 | 1399.51 | Y | Y | Y | Y | Y | Y | (Hex)_3_ + (Man)_3_(GlcNAc)_2_ | 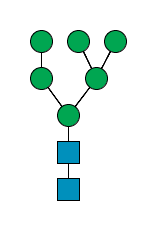 |
| 1b | 28.57 |  |  |  | Y | Y | Y | Y | Y | Y |  |  |
| 1c | 32.26 |  |  |  | Y | Y | Y | Y | Y | Y |  |  |
| 1d | 32.95 |  |  |  | Y | Y | Y | Y | Y | Y |  |  |
| 2a | 29.24 | 489.19 | 733.29 | 1465.57 | Y | Y | Y | Y | Y | N | (HexNAc)_2_ (Deoxyhexose)_1_ + (Man)_3_(GlcNAc)_2_ | 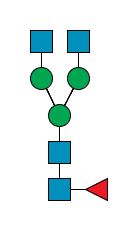 |
| 2b | 32.36 |  |  |  | Y | Y | Y | Y | Y | Y |  |  |
| 2c | 36.72 |  |  |  | Y | Y | Y | Y | Y | Y |  |  |
| 3a | 33.04 | 494.53 | 741.28 | 1481.56 | Y | N | Y | Y | N | N | (Hex)_1_ (HexNAc)_2_ + (Man)_3_(GlcNAc)_2_ | 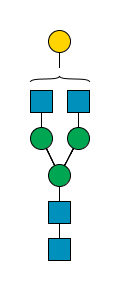 |
| 3b | 33.81 |  |  |  | Y | N | Y | Y | Y | Y |  |  |
| 4a | 28.50 | 521.19 | 781.28 | 1561.56 | Y | Y | Y | N | Y | Y | (Hex)_4_ + (Man)_3_(GlcNAc)_2_ | 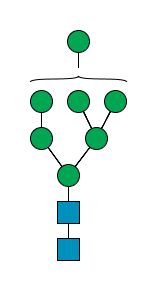 |
| 4b | 29.43 |  |  |  | N | N | N | N | Y | Y |  |  |
| 4c | 32.02 |  |  |  | Y | Y | Y | Y | Y | Y |  |  |
| 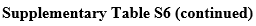4d | 32.33 |  |  |  | Y | Y | Y | Y | Y | Y |  |  |
| 4e | 32.96 |  |  |  | Y | Y | Y | Y | Y | Y |  |  |
| 5a | 39.31 | 523.86 | 785.29 | 1569.58 | Y | Y | Y | Y | Y | Y | (Hex)_1_ (HexNAc)_1_ (NeuAc)_1_ + (Man)_3_(GlcNAc)_2_ | 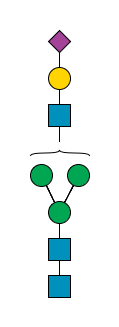 |
| 5b | 39.70 |  |  |  | Y | Y | Y | Y | Y | Y |  |  |
| 5c | 41.65 |  |  |  | Y | Y | Y | Y | Y | Y |  |  |
| 6a | 30.74 | 534.87 | 801.80 | 1602.59 | Y | Y | Y | N | Y | Y | (Hex)_3_ (HexNAc)_1_ + (Man)_3_(GlcNAc)_2_ | 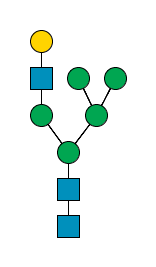 |
| 6b | 34.78 |  |  |  | Y | Y | Y | Y | Y | Y |  |  |
| 7a | 31.13 | 543.21 | 814.31 | 1627.62 | Y | Y | Y | Y | Y | Y | (Hex)_1_ (HexNAc)_2_ (Deoxyhexose)_1_ + (Man)_3_(GlcNAc)_2_ | 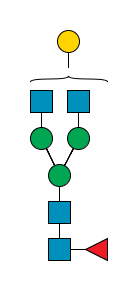 |
| 7b | 32.00 |  |  |  | N | N | N | Y | N | N |  |  |
| 7c | 32.57 |  |  |  | Y | Y | Y | Y | Y | Y |  |  |
| 7d | 33.34 |  |  |  | N | N | Y | Y | N | N |  |  |
| 7e | 34.12 |  |  |  | Y | Y | Y | Y | N | N |  |  |
| 7f | 34.50 |  |  |  | Y | Y | Y | Y | Y | Y |  |  |
| 7g | 38.45 |  |  |  | Y | Y | Y | Y | Y | Y |  |  |
| 7h | 38.93 |  |  |  | Y | Y | Y | Y | Y | Y |  |  |
| 8a | 24.55 | 548.54 | 822.31 | 1643.62 | Y | Y | Y | Y | Y | Y | (Hex)_2_ (HexNAc)_2_ + (Man)_3_(GlcNAc)_2_ | 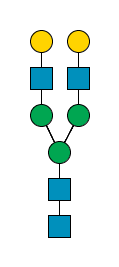 |
| 8b | 31.61 |  |  |  | N | N | Y | Y | Y | Y |  |  |
| 8c | 32.63 |  |  |  | Y | N | Y | Y | Y | Y |  |  |
| 8d | 35.86 |  |  |  | Y | Y | Y | Y | Y | Y |  |  |
| 9a | 25.54 | 556.89 | 834.83 | 1668.65 | N | N | Y | Y | Y | N | (HexNAc)_3_ (Deoxyhexose)_1_ + (Man)_3_(GlcNAc)_2_ | 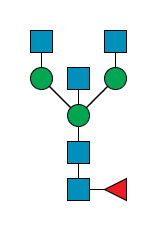 |
| 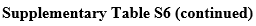9b | 26.47 |  |  |  | N | N | Y | Y | Y | N |  |  |
| 9c | 29.13 |  |  |  | Y | Y | Y | Y | Y | Y |  |  |
| 10a | 40.83 | 572.55 | 858.32 | 1715.64 | Y | Y | Y | Y | Y | Y | (Hex)_1_ (HexNAc)_1_ (Deoxyhexose)_1_ (NeuAc)_1_ + (Man)_3_(GlcNAc)_2_ | 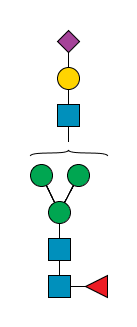 |
| 10b | 41.92 |  |  |  | Y | Y | Y | Y | Y | Y |  |  |
| 10c | 42.46 |  |  |  | Y | Y | Y | Y | Y | Y |  |  |
| 10d | 46.32 |  |  |  | Y | Y | Y | Y | Y | Y |  |  |
| 11a | 27.83 | 575.21 | 862.31 | 1723.61 | Y | Y | Y | Y | Y | Y | (Hex)_5_ + (Man)_3_(GlcNAc)_2_ | 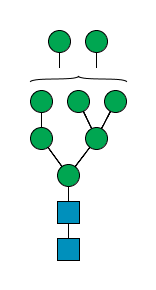 |
| 11b | 28.31 |  |  |  | Y | Y | Y | Y | Y | Y |  |  |
| 11c | 29.23 |  |  |  | Y | Y | Y | Y | Y | Y |  |  |
| 11d | 31.93 |  |  |  | Y | Y | Y | Y | Y | Y |  |  |
| 11e | 32.58 |  |  |  | Y | Y | Y | Y | Y | Y |  |  |
| 12a | 39.65 | 597.23 | 895.34 | 1789.67 | Y | Y | Y | Y | Y | Y | (Hex)_2_ (HexNAc)_2_ (Deoxyhexose)_1_ + (Man)_3_(GlcNAc)_2_ | 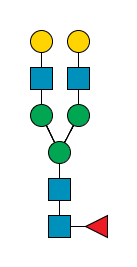 |
| 12b | 40.74 |  |  |  | Y | Y | Y | Y | Y | Y |  |  |
| 13a | 30.27 | 610.90 | 915.85 | 1830.70 | Y | Y | Y | Y | Y | Y | (Hex)_1_ (HexNAc)_3_ (Deoxyhexose)_1_ + (Man)_3_(GlcNAc)_2_ | 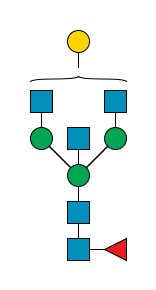 |
| 13b | 30.91 |  |  |  | Y | Y | Y | Y | Y | Y |  |  |
| 13c | 34.43 |  |  |  | Y | Y | Y | Y | Y | Y |  |  |
| 13d | 39.34 |  |  |  | Y | Y | Y | Y | Y | Y |  |  |
| 14a | 27.80 | 629.23 | 943.34 | 1885.67 | Y | Y | Y | Y | Y | Y | (Hex)_6_ + (Man)_3_(GlcNAc)_2_ | 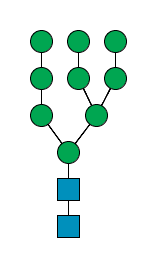 |
| 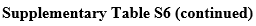14b | 28.55 |  |  |  | N | N | N | Y | Y | N |  |  |
| 14c | 29.25 |  |  |  | Y | Y | Y | Y | Y | Y |  |  |
| 14d | 32.93 |  |  |  | Y | Y | Y | Y | Y | Y |  |  |
| 15a | 31.35 | 631.90 | 947.35 | 1893.68 | N | N | Y | Y | N | N | (Hex)_3_ (HexNAc)_1_ (NeuAc)_1_ + (Man)_3_(GlcNAc)_2_ | 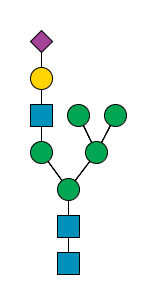 |
| 15b | 34.75 |  |  |  | Y | Y | Y | N | Y | N |  |  |
| 15c | 36.51 |  |  |  | N | N | Y | Y | N | N |  |  |
| 15d | 38.21 |  |  |  | N | N | Y | N | Y | Y |  |  |
| 15e | 40.05 |  |  |  | Y | Y | Y | Y | Y | Y |  |  |
| 15f | 40.53 |  |  |  | N | N | Y | Y | Y | Y |  |  |
| 15g | 40.88 |  |  |  | Y | Y | Y | Y | Y | Y |  |  |
| 15h | 42.00 |  |  |  | Y | Y | Y | Y | Y | Y |  |  |
| 16a | 35.80 | 645.58 | 967.86 | 1934.71 | Y | Y | Y | Y | Y | Y | (Hex)_2_ (HexNAc)_2_ (NeuAc)_1_ + (Man)_3_(GlcNAc)_2_ | 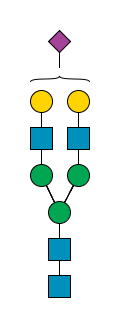 |
| 16b | 36.78 |  |  |  | Y | Y | Y | Y | Y | Y |  |  |
| 16c | 38.69 |  |  |  | Y | Y | Y | Y | Y | Y |  |  |
| 16d | 40.05 |  |  |  | Y | Y | Y | Y | Y | Y |  |  |
| 17a | 32.63 | 664.92 | 996.88 | 1992.75 | Y | Y | Y | Y | Y | Y | (Hex)_2_ (HexNAc)_3_ (Deoxyhexose)_1_ + (Man)_3_(GlcNAc)_2_ | 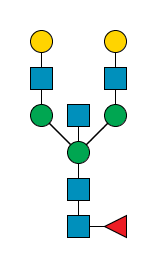 |
| 17b | 36.61 |  |  |  | Y | Y | Y | Y | Y | Y |  |  |
| 17c | 40.97 |  |  |  | Y | Y | Y | N | Y | N |  |  |
| 18a | 30.13 | 683.25 | 1024.36 | 2047.72 | Y | Y | Y | Y | Y | Y | (Hex)_7_ + (Man)_3_(GlcNAc)_2_ | 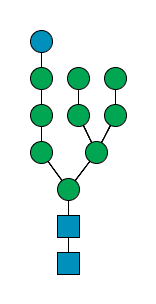 |
| 18b | 31.41 |  |  |  | Y | Y | Y | Y | Y | Y |  |  |
| 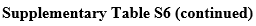18c | 34.92 |  |  |  | Y | Y | Y | Y | Y | Y |  |  |
| 19a | 38.44 | 694.26 | 1040.89 | 2080.77 | Y | Y | Y | Y | Y | Y | (Hex)_2_ (HexNAc)_2_ (Deoxyhexose)_1_ (NeuAc)_1_ + (Man)_3_(GlcNAc)_2_ | 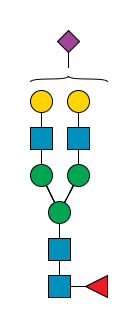 |
| 19b | 39.21 |  |  |  | Y | Y | Y | Y | Y | Y |  |  |
| 19c | 40.17 |  |  |  | Y | Y | Y | Y | Y | Y |  |  |
| 19d | 40.97 |  |  |  | Y | Y | Y | Y | Y | Y |  |  |
| 19e | 41.85 |  |  |  | Y | Y | Y | Y | Y | Y |  |  |
| 19f | 42.55 |  |  |  | Y | Y | Y | Y | Y | Y |  |  |
| 19g | 43.36 |  |  |  | Y | Y | Y | Y | Y | Y |  |  |
| 20a | 38.34 | 761.95 | 1142.43 | 2283.85 | Y | Y | Y | Y | Y | Y | (Hex)_2_ (HexNAc)_3_ (Deoxyhexose)_1_ (NeuAc)_1_ + (Man)_3_(GlcNAc)_2_ | 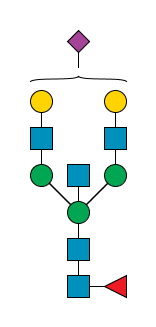 |
| 20b | 39.39 |  |  |  | Y | Y | Y | Y | Y | Y |  |  |
| 20c | 40.07 |  |  |  | Y | Y | Y | Y | Y | Y |  |  |
| 20d | 40.97 |  |  |  | Y | Y | Y | Y | Y | Y |  |  |
| 20e | 41.18 |  |  |  | Y | Y | Y | Y | Y | Y |  |  |
| 21 | 40.38 | 709.90 | 1064.35 | 2127.69 | Y | Y | Y | Y | Y | Y | (Hex)_7_ (Phos)_1_ + (Man)_3_(GlcNAc)_2_ | 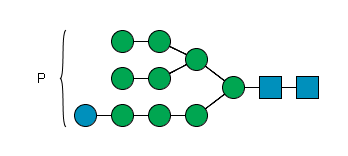 |
| 22a | 39.68 | 742.60 | 1113.41 | 2225.81 | Y | Y | Y | Y | Y | Y | (Hex)_2_ (HexNAc)_2_ (NeuAc)_2_ + (Man)_3_(GlcNAc)_2_ | 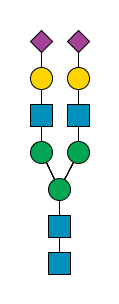 |
| 22b | 39.91 |  |  |  | Y | Y | Y | Y | Y | Y |  |  |
| 22c | 41.23 |  |  |  | Y | Y | Y | Y | Y | Y |  |  |
| 22d | 41.67 |  |  |  | Y | Y | Y | Y | Y | Y |  |  |
| 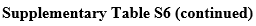22e | 42.67 |  |  |  | Y | Y | Y | Y | Y | Y |  |  |
| 22f | 43.31 |  |  |  | Y | Y | N | Y | Y | Y |  |  |
| 22g | 44.16 |  |  |  | N | Y | Y | Y | Y | Y |  |  |
| 22h | 45.81 |  |  |  | Y | Y | Y | Y | Y | Y |  |  |
| 23a | 40.46 | 791.29 | 1186.44 | 2371.86 | N | N | Y | Y | Y | Y | (Hex)_2_ (HexNAc)_2_ (Deoxyhexose)_1_ (NeuAc)_2_ + (Man)_3_(GlcNAc)_2_ | 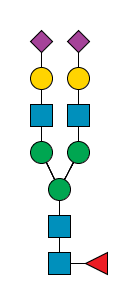 |
| 23b | 40.62 |  |  |  | N | Y | Y | Y | Y | Y |  |  |
| 23c | 42.01 |  |  |  | Y | N | Y | Y | Y | Y |  |  |
| 23d | 42.52 |  |  |  | Y | Y | Y | Y | Y | Y |  |  |
| 23e | 43.44 |  |  |  | Y | Y | Y | Y | Y | Y |  |  |
| 23f | 43.85 |  |  |  | Y | Y | Y | Y | Y | Y |  |  |
| 23g | 44.55 |  |  |  | Y | Y | Y | Y | N | Y |  |  |
| 23h | 44.87 |  |  |  | Y | Y | Y | Y | Y | Y |  |  |
| 23i | 45.62 |  |  |  | Y | Y | Y | Y | Y | Y |  |  |
| 23j | 46.12 |  |  |  | Y | Y | Y | Y | Y | Y |  |  |
| 23k | 47.06 |  |  |  | Y | Y | Y | Y | Y | Y |  |  |
| 23l | 51.73 |  |  |  | N | Y | Y | N | Y | Y |  |  |
| 24a | 40.53 | 815.97 | 1223.45 | 2445.90 | Y | Y | Y | Y | Y | Y | (Hex)_3_ (HexNAc)_3_ (Deoxyhexose)_1_ (NeuAc)_1_ + (Man)_3_(GlcNAc)_2_ | 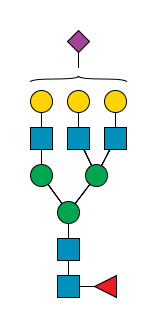 |
| 24b | 41.03 |  |  |  | Y | Y | Y | Y | Y | Y |  |  |
| 24c | 41.84 |  |  |  | Y | Y | Y | Y | Y | Y |  |  |
| 24d | 42.30 |  |  |  | Y | Y | Y | Y | Y | Y |  |  |
| 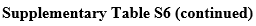24e | 43.02 |  |  |  | Y | Y | Y | Y | Y | Y |  |  |
| 24f | 43.42 |  |  |  | Y | N | Y | Y | Y | Y |  |  |
| 24g | 44.22 |  |  |  | Y | Y | Y | Y | Y | Y |  |  |
| 24h | 45.13 |  |  |  | Y | Y | Y | N | Y | Y |  |  |
| 25a | 31.76 | 413.16 | 619.23 | 1237.46 | Y | Y | Y | Y | Y | Y | (Hex)_2_ + (Man)_3_(GlcNAc)_2_ | 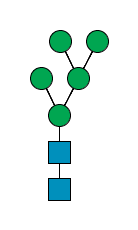 |
| 25b | 32.13 |  |  |  | Y | Y | Y | Y | Y | Y |  |  |
| 25c | 32.86 |  |  |  | Y | Y | Y | Y | Y | Y |  |  |
| 25d | 37.82 |  |  |  | Y | Y | Y | Y | Y | Y |  |  |
| 26a | 38.15 | 858.99 | 1287.98 | 2574.94 | Y | Y | Y | Y | N | N | (Hex)_2_ (HexNAc)_3_ (Deoxyhexose)_1_ (NeuAc)_2_ + (Man)_3_(GlcNAc)_2_ | 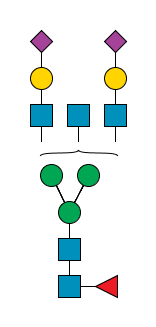 |
| 26b | 41.28 |  |  |  | Y | Y | Y | Y | Y | Y |  |  |
| 26c | 41.95 |  |  |  | Y | Y | Y | Y | N | N |  |  |
| 26d | 44.87 |  |  |  | Y | Y | Y | Y | Y | N |  |  |
